# Deep Learning Analysis of CBCT Images for Periodontal Disease: Phenotype-Level Concordance with Independent Transcriptomic and Microbiome Datasets

**DOI:** 10.3390/dj13120578

**Published:** 2025-12-03

**Authors:** Ștefan Lucian Burlea, Călin Gheorghe Buzea, Florin Nedeff, Diana Mirilă, Valentin Nedeff, Maricel Agop, Lăcrămioara Ochiuz, Adina Oana Armencia

**Affiliations:** 1 Dentoalveolar Surgery, Faculty of Medicine, University of Medicine and Pharmacy “Grigore T. Popa” Iași, 700115 Iași, Romania; stefan.burlea@umfiasi.ro; 2 National Institute of Research and Development for Technical Physics—IFT Iași, 700050 Iași, Romania; calinb2003@yahoo.com; 3 Clinical Emergency Hospital “Prof. Dr. Nicolae Oblu” Iași, 700309 Iași, Romania; 4 Department of Environmental Engineering, Mechanical Engineering and Agritourism, Faculty of Engineering, “Vasile Alecsandri” University of Bacău, 600115 Bacău, Romania; florin_nedeff@ub.ro (F.N.); vnedeff@ub.ro (V.N.); m.agop@yahoo.com (M.A.); 5 Faculty of Pharmacy, University of Medicine and Pharmacy “Grigore T. Popa” Iași, 700115 Iași, Romania; lacramioara.ochiuz@umfiasi.ro; 6 Discipline of Oral and Community Health, Department I–Surgical Sciences, Faculty of Dental Medicine, University of Medicine and Pharmacy “Grigore T. Popa” Iași, 700115 Iași, Romania; adina.armencia@umfiasi.ro

**Keywords:** periodontitis, cone-beam computed tomography, deep learning, gene expression profiling, microbiota, machine learning

## Abstract

Background: Periodontitis is a common inflammatory disease characterized by progressive loss of alveolar bone. Cone-beam computed tomography (CBCT) can visualize 3D periodontal bone defects, but its interpretation is time-consuming and examiner-dependent. Deep learning may support standardized CBCT assessment if performance and biological relevance are adequately characterized. Methods: We used the publicly available MMDental dataset (403 CBCT volumes from 403 patients) to train a 3D ResNet-18 classifier for binary discrimination between periodontitis and healthy status based on volumetric CBCT scans. Volumes were split by subject into training (n = 282), validation (n = 60), and test (n = 61) sets. Model performance was evaluated using area under the receiver operating characteristic curve (AUROC), area under the precision–recall curve (AUPRC), and calibration metrics with 95% bootstrap confidence intervals. Grad-CAM saliency maps were used to visualize the anatomical regions driving predictions. To explore phenotype-level biological concordance, we analyzed an independent gingival transcriptomic cohort (GSE10334, n ≈ 220 arrays after quality control) and an independent oral microbiome cohort based on 16S rRNA amplicon sequencing, using unsupervised clustering, differential expression/abundance testing, and pathway-level summaries. Results: On the held-out CBCT test set, the model achieved an AUROC of 0.729 (95% CI: 0.599–0.850) and an AUPRC of 0.551 (95% CI: 0.404–0.727). At a high-sensitivity operating point (sensitivity 0.95), specificity was 0.48, yielding an overall accuracy of 0.62. Grad-CAM maps consistently highlighted the alveolar crest and furcation regions in periodontitis cases, in line with expected patterns of bone loss. In the transcriptomic cohort, inferred periodontitis samples showed up-regulation of inflammatory and osteoclast-differentiation pathways and down-regulation of extracellular-matrix and mitochondrial programs. In the microbiome cohort, disease-associated samples displayed a dysbiotic shift with enrichment of classic periodontal pathogens and depletion of health-associated commensals. These omics patterns are consistent with an inflammatory–osteolytic phenotype that conceptually aligns with the CBCT-defined disease class. Conclusions: This study presents a proof-of-concept 3D deep learning model for CBCT-based periodontal disease classification that achieves moderate discriminative performance and anatomically plausible saliency patterns. Independent transcriptomic and microbiome analyses support phenotype-level biological concordance with the imaging-defined disease class, but do not constitute subject-level multimodal validation. Given the modest specificity, single-center imaging source, and inferred labels in the omics cohorts, our findings should be interpreted as exploratory and hypothesis-generating. Larger, multi-center CBCT datasets and prospectively collected paired imaging–omics cohorts are needed before clinical implementation can be considered.

## 1. Introduction

Periodontitis is a chronic multifactorial disease characterized by inflammation, destruction of periodontal tissues, and progressive loss of alveolar bone, ultimately leading to tooth loss if untreated. Beyond localized tissue destruction, periodontitis has systemic implications, with evidence linking oral bone loss to osteoporotic processes and systemic skeletal changes [1]. Accurate diagnosis and staging are crucial for effective treatment planning, yet current clinical standards—probing pocket depth (PPD), clinical attachment loss (CAL), and conventional radiography—are limited by examiner variability and by their inability to capture the full three-dimensional extent of bone loss [2,3]. These limitations have motivated the use of advanced imaging and, more recently, AI-assisted diagnostics in periodontology [4], aligning with broader trends in medicine where artificial intelligence is increasingly used to support diagnostic accuracy and decision-making [5].

**Current diagnostic modalities.** Periodontal evaluation traditionally relies on clinical probing measurements (PPD, CAL, and bleeding on probing) and two-dimensional radiographic imaging (periapical, bitewing, and panoramic radiographs). While these approaches form the clinical standard, they suffer from examiner-dependent variability and projection-related distortions that limit the detection of vertical defects, early cortical plate loss, furcation anatomy, and the true spatial extent of bone destruction.

**Epidemiology of periodontitis.** Periodontitis is one of the most prevalent chronic inflammatory diseases worldwide. Mild–moderate forms affect nearly 50% of adults, while severe periodontitis affects about 10% of the global population—representing a major public-health burden driven by chronicity, functional impairment, and associations with systemic diseases. This high prevalence underscores the need for early, reproducible, and anatomically precise diagnostic tools capable of detecting clinically relevant bone changes.

**Role of CBCT in periodontal diagnosis.** Cone-beam computed tomography (CBCT) provides high-resolution volumetric visualization of dental and periodontal structures, enabling more accurate assessment of bone morphology, furcation involvement, and volumetric changes compared with 2D imaging [4,6,7,8]. Automated tools for tooth and jaw delineation in CBCT have improved reproducibility and throughput [9]. The integration of artificial intelligence (AI) and deep learning (DL) further enhances CBCT interpretation: convolutional neural networks have shown promising results in automating the detection of periodontal bone loss, staging periodontitis, and identifying furcation involvement [6,8], and recent CBCT–AI work has enabled direct volumetric detection of tooth status and bone-defect patterns [9]. Systematic reviews highlight rapid progress in dental AI, while also emphasizing persistent limitations such as small single-center datasets, heterogeneous imaging protocols, lack of external validation, and limited biological interpretability [2,10,11].

Clinically, expert radiographic performance and CBCT-AI systems typically fall within an AUROC range of **0.70–0.80** [6,7,8,9,10,11], indicating that deep learning models in this domain should be viewed as **adjunctive decision-support tools**, not replacements for expert assessment. Interpretability methods such as Grad-CAM and SHAP have been increasingly adopted to visualize model attention maps in CBCT, aiming to improve clinician trust and assess anatomical plausibility [12]. However, most CBCT–AI studies remain limited to imaging-level analyses and do not connect imaging phenotypes to underlying biological processes.

**Biological context: transcriptomics and microbiome.** Periodontal disease has also been extensively characterized at the molecular and microbial levels. Transcriptomic studies—such as GEO dataset GSE10334—report robust differences between diseased and healthy gingival tissues, highlighting immune activation, extracellular-matrix remodeling, and inflammatory signaling pathways [13,14,15]. Oral-microbiome studies consistently describe dysbiosis in periodontitis, with increased abundance of classical pathogens (Porphyromonas gingivalis, Treponema denticola, and Tannerella forsythia) and depletion of health-associated commensals [16,17]. Functional analyses implicate these microbial shifts in tissue-destructive host–microbe interactions [18,19], while longitudinal multi-omic work demonstrates coordinated host and microbial activity during disease progression [20]. Yet, these molecular and microbial datasets are almost always independent of imaging cohorts, leaving the relationship between CBCT-visible structural loss and omics-defined biology largely unexplored.

**Need for cross-modal integration.** Current imaging-based DL models rarely link their predictions to omics-level information, while omics studies seldom relate their findings to CBCT-detectable structural changes. This disconnect limits the biological interpretation of imaging biomarkers and the development of mechanistically grounded AI models. Systems-biology frameworks in periodontitis emphasize the value of multimodal integration [21], and recent reviews explicitly call for biologically interpretable AI and cross-modal approaches in dental research [22]. Because paired imaging–omics datasets are rare, phenotype-level triangulation across independent cohorts—while indirect—remains a practical approach to evaluating whether an imaging-defined disease phenotype aligns with established biological signatures.

**Rationale for the present study.** This study uses a two-step, phenotype-level approach. First, we develop and evaluate a 3D deep learning model based on CBCT volumes from the MMDental dataset to classify periodontal disease, including analyses of discrimination, calibration, and Grad-CAM interpretability. Second, we contextualize the imaging-defined phenotype using independent gingival transcriptomic (GSE10334) and oral-microbiome amplicon sequence variant (ASV) datasets. In these datasets—where no CBCT is available—we use unsupervised clustering and downstream analyses to ask whether inferred “diseased” vs. “healthy-like” phenotypes recapitulate gene expression and microbial patterns characteristic of an inflammatory–osteolytic periodontal state. Because the cohorts are non-overlapping, this triangulation evaluates **phenotype-level concordance** rather than subject-level multimodal integration and is framed as exploratory and hypothesis-generating.

To our knowledge, no prior study has developed a CBCT-based deep learning model for periodontal diagnosis and then examined the same disease phenotype across transcriptomic and microbiome layers in independent cohorts. Prior research typically focuses on either imaging or omics in isolation [22]. Our study addresses this gap through phenotype-level triangulation, linking CBCT imaging, gingival gene expression, and oral-microbiome composition.

### Study Aims and Contributions

This study was designed to address the gap between CBCT-derived structural phenotypes and the molecular and microbial signatures of periodontal disease. We formulated the following hypotheses:

1. **CBCT-based deep learning for disease discrimination:**
  A 3D convolutional neural network trained solely on CBCT volumes from the MMDental dataset can discriminate periodontal disease status with performance comparable to other imaging-based diagnostic approaches and can localize anatomically plausible regions of interest using Grad-CAM.

2. **Transcriptomic correspondence:**
  The imaging-defined disease phenotype will correspond, at the cohort level, to gingival transcriptomic signatures enriched for inflammation, immune activation, and extracellular-matrix remodeling in the independent GSE10334 dataset.

3. **Microbiome correspondence:**
  The same phenotype will correspond, at the cohort level, to a dysbiotic oral-microbiome community enriched in anaerobic and proteolytic taxa classically associated with periodontitis in an independent 16S rRNA cohort.

Methodologically, this study contributes the following:

- A reproducible CBCT preprocessing and 3D CNN training pipeline with transparent reporting of model selection and uncertainty;
- A label-inference and ambiguity-filtering strategy for legacy microarray series matrices lacking explicit clinical labels;
- A compositionality-aware microbiome workflow (centered log-ratio transformation, Bray–Curtis/Aitchison distances, PERMANOVA, differential abundance testing, and genus-level elastic-net prediction);
- A phenotype-level triangulation framework synthesizing imaging, transcriptomic, and microbiome evidence across non-overlapping cohorts.

These components aim to move CBCT-based AI beyond pure prediction toward biologically interpretable, externally contextualized phenotypes in periodontology.

**Overview of the paper.** Section 2 (Materials and Methods) describes the CBCT dataset, preprocessing, model architectures, and training/evaluation procedures, as well as the transcriptomic and microbiome analysis pipelines. Section 3 (Results) presents the imaging and omics findings, and Section 4 (Discussion) interprets these results, discusses limitations, and outlines future directions, followed by conclusions in Section 5.

## 2. Materials and Methods

### 2.1. Study Rationale

The purpose of this study is to evaluate whether a 3D deep learning model trained on CBCT volumes can identify a clinically meaningful periodontal disease phenotype and whether this imaging-defined phenotype shows biological consistency when examined in independent transcriptomic and microbiome cohorts. The MMDental dataset was selected because it represents one of the few publicly available CBCT collections with subject-level clinical labels suitable for AI model development. Given the lack of paired imaging–omics datasets in the literature, our analysis focuses on phenotype-level concordance across independent cohorts rather than subject-level multimodal fusion. Accordingly, the study is designed as a proof-of-concept investigation intended to assess technical feasibility, biological plausibility, and the potential value of cross-modal contextualization.

### 2.2. Dataset

This study used cone-beam computed tomography (CBCT) scans from the publicly available MMDental dataset (Wang et al., 2025 [23]). MMDental is a peer-reviewed multimodal dental imaging dataset comprising 3D CBCT volumes and accompanying clinical records from 660 patients (age 5–86 years; 51.1% male), collected at Hangzhou Dental Hospital under approval of the Medical Ethics Committee of Lishui University (Approval No. 2022YR014). All data were anonymized and released under a CC-BY license. CBCT scans were acquired on HiRes 3D-Plus and Boen Oral & Maxillofacial CBCT systems under standardized protocols (90–100 kVp, 3–9 mA, 0.25 mm slice thickness, 16 cm × 10 cm FOV).

Because MMDental is an existing open-access dataset and no new CBCT examinations were acquired, we acknowledge that this limits the novelty and external generalizability of the study. Accordingly, the present work is framed as a single-center proof-of-concept analysis.

All volumes were converted to NIfTI format (nii). After excluding incomplete or corrupted files based on visual inspection and header-integrity checks, 403 subjects remained and were split by independent patients into training (n = 282), validation (n = 60), and test (n = 61) subsets to avoid data leakage.

**Classification target.** The primary task was binary discrimination between periodontitis and healthy status. Although the dataset includes staging labels (Stages I–IV), these were collapsed to binary due to (i) substantial class imbalance across stages, (ii) limited inter-rater reliability reported for fine staging in public periodontal datasets, and (iii) increased misclassification risk when training 3D CNNs on small per-class sample sizes. Binary labeling, therefore, provided a more statistically stable and reproducible training signal. We acknowledge that this reduces diagnostic granularity and highlight this limitation in the Discussion.

**Label source and verification.** Ground-truth labels were extracted from *medical_records.csv*, which includes clinician adjudication based on chart review. When available, probing measurements (PPD/CAL) and radiographic notes (bone loss patterns, furcation involvement) informed the binary label. Basic quality-control checks were performed to ensure internal consistency, including verification of patient–scan matching, detection of duplicated entries, and identification of missing or ambiguous labels. As the original clinical charts cannot be accessed, some degree of label noise cannot be excluded; this is recognized as a potential bias.

**Inclusion/Exclusion.** We excluded scans with severe motion artifacts, truncated fields of view that omitted the alveolar crest, corrupted headers (inconsistent affine matrices or voxel spacing), or ambiguous labels. Scans containing orthodontic hardware were retained if artifacts did not affect the target anatomical regions.

**External validation.** No multi-center or independent CBCT dataset was available for external testing. Therefore, all results reflect performance on a single-center dataset and should be interpreted accordingly. The need for multi-center external validation is emphasized in the Limitations.

A summary of demographic and acquisition characteristics is provided in Table 1.

### 2.3. Image Preprocessing

To standardize spatial context while preserving diagnostically relevant periodontal anatomy, all CBCT volumes were converted to NIfTI format and resampled to an isotropic 96 × 96 × 96 voxel grid. The cube size was chosen based on preliminary experiments comparing 64^3^, 96^3^, and 128^3^ resolutions, balancing anatomical detail, GPU memory constraints, and model stability. Prior CBCT-based 3D CNN studies commonly adopt patch sizes in the 64–128^3^ range; in our tests, 96^3^ provided the most favorable trade-off between structural fidelity and training convergence. Although downsampling inevitably reduces resolution, we explicitly address this limitation in the Discussion.

**Resampling.** Volumes were resampled using tri-linear interpolation for image data and nearest-neighbor interpolation for any label masks. Original physical voxel spacing was retained in metadata to enable sensitivity checks.

**Intensity normalization and scanner harmonization.** Because CBCT intensities are not standardized (unlike Hounsfield units), each volume underwent per-volume z-score normalization:

(1) $$x^{\prime }=\frac{x-\mu_{x}}{\sigma_{x}+10^{-6}}$$

where $\mu_{x}$ and $\sigma_{x}$ are the mean and standard deviation of the current volume. The MMDental dataset includes scans from two related CBCT systems (HiRes 3D-Plus and Boen). To mitigate scanner-related intensity variability, we applied a harmonization pipeline consisting of the following:

(i) Resampling all scans to uniform voxel spacing;

(ii) Per-volume z-score standardization.

We additionally tested slice-level histogram matching between scanner types but did not adopt it due to amplification of noise in a subset of scans; scanner heterogeneity, therefore, remains a limitation.

**Cropping and centering.** A fixed 96^3^ cube was centered on the dental arches using a heuristic bounding box derived from the largest non-air connected component (intensity thresholding + morphological closing), ensuring consistent inclusion of the alveolar crest and furcation regions.

**Data augmentation.** To reduce overfitting and improve generalization, we applied a set of realistic 3D augmentations during training:

- Random flips along all three axes (*p* = 0.5 each);
- Random in-plane 90° rotations (k ϵ {0, 1, 2, 3});
- Small random rotations (±10°);
- Random scaling (±10%);
- Low-amplitude Gaussian noise addition;
- Mild intensity jitter.

Augmentations were applied probabilistically to each training volume. More aggressive elastic deformations were avoided to preserve subtle trabecular and cortical bone patterns. Overfitting was monitored using validation loss, AUROC, and calibration curves, with early stopping, dropout, and weight decay included in the training procedure.

**Archival.** The final preprocessed dataset (~1.2 GB) was archived and split into three parts for compatibility with cloud platforms (Google Colab) to ensure fully reproducible data loading and preprocessing.

### 2.4. Model Architectures

Given the modest size of the available dataset (n = 403), we evaluated **lightweight 3D CNN architectures** chosen to balance expressive capacity with overfitting risk. Three variants were tested:

- 3D ResNet-18 (R3D-18)—baseline volumetric convolutional network.
- R(2+1)D-18—factorized convolutions (2D spatial + 1D depth) initialized from Kinetics-400 pretraining.
- R3D-18 with exponential moving average (EMA)—stability-enhanced variant.

All models were adapted for single-channel CBCT by replacing the initial 3-channel convolution with a 1-channel kernel. The classification head consisted of global average pooling followed by a fully connected layer with sigmoid activation.

Preliminary tests with deeper 3D architectures (ResNet-34/50) showed substantial overfitting despite regularization, so 18-layer variants were used for the final models.

### 2.5. Training Procedure

All models were trained on Google Colab T4 GPUs using the following:

- Optimizer: AdamW (learning rate = 1 × 10^−4^, weight decay = 1 × 10^−4^).
- Loss: BCEWithLogitsLoss with class weighting (pos_weight ≈ 3.0).
- Scheduler: cosine decay with 5-epoch warm-up.
- Batch size: 4.
- Regularization: dropout (*p* = 0.3), automatic mixed-precision (AMP), and gradient clipping (norm ≤ 1).
- Early stopping: 10–12 epochs based on validation AUROC.

Training was performed using a **single fixed subject-level split** (282 train/60 validation/61 test). No cross-validation was performed due to the computational cost of 3D CNN training and the lack of multi-center datasets. The study is, therefore, framed as a **single-center proof-of-concept**, and the need for multi-center validation is emphasized.

Ablation variants (EMA, R(2+1)D, focal loss, partial freezing, and test-time augmentation) were explored but did not consistently improve performance over the baseline R3D-18.

### 2.6. Evaluation Metrics and Statistical Analysis

Model performance on the held-out test set was evaluated using the following:

- AUROC (primary metric).
- AUPRC.
- Accuracy.
- Sensitivity.
- Specificity.
- F1-score.

**Confidence intervals**

Statistical uncertainty was quantified using **95% confidence intervals** derived from **2000-sample nonparametric bootstrapping** of test predictions. The wide intervals reflect expected variability for a dataset of this size and are acknowledged as a limitation.

**Calibration analysis**

Calibration was assessed using the **Brier score**, **Expected Calibration Error (ECE; 15 bins)**, and reliability diagrams. These analyses are used descriptively and not to justify clinical deployment.

**Operating points**

Metrics are reported at the following:

- Threshold = 0.5;
- The Youden J index;
- The F1-maximizing threshold.

**External validation**

No independent multi-center CBCT dataset is currently available for periodontal disease; therefore, all results represent **single-center generalization**.

**Reproducibility**

Random seeds (Python 3.12.2/NumPy 2.0.2/PyTorch 12.6) were fixed, and all preprocessing and training scripts are provided in the supplementary repository.

### 2.7. Model Interpretability (Saliency)

Model interpretability was assessed using Grad-CAM applied to the final convolutional block of the trained 3D ResNet-18 model. Grad-CAM heatmaps were obtained by backpropagating the gradient of the predicted class score to the last convolutional feature maps, followed by ReLU activation and tri-linear upsampling to the CBCT input resolution.

For visualization, heatmaps were min–max normalized on a per-volume basis and overlaid on axial, coronal, and sagittal slices. These qualitative maps were inspected to determine whether the classifier attended to anatomically plausible periodontal regions, such as the alveolar crest and furcation areas.

Because the MMDental dataset does not provide voxel-level expert annotations or region-specific segmentation masks, no quantitative evaluation (e.g., Dice overlap, ROI-wise saliency quantification) could be performed. We also note that Grad-CAM may highlight image edges or density gradients rather than true pathological structures; therefore, saliency results are interpreted exploratorily. Future work with annotated CBCT datasets will be required to establish quantitative interpretability benchmarks.

### 2.8. External Biological Contextualization—Transcriptomics (GSE10334)

Because the CBCT cohort does not include paired molecular measurements, analyses of GSE10334 were performed as exploratory, population-level contextualization rather than biological validation of the imaging classifier.

#### 2.8.1. Data Ingestion and Normalization

The GSE10334 Affymetrix HG-U133 Plus 2.0 series matrix (GPL570) was parsed into a probe × sample matrix. All available !Sample_* header fields were aggregated into per-sample metadata. Expression values were log-checked and quantile-normalized across samples to reduce technical variability.

#### 2.8.2. Unsupervised Label Inference and Ambiguity Filtering

Because GSE10334 does not include labels aligned with the imaging cohort, disease status was inferred using a combination of metadata parsing and unsupervised clustering:

1. Metadata fields were scanned for diagnostic keywords (“periodontitis”, “healthy”, and “control”) to obtain an initial tentative label.
2. When metadata were inconsistent or incomplete, PCA followed by k-means clustering (k = 2) on the normalized expression matrix was used to identify two major transcriptomic groups. Cluster naming was guided by the majority keyword match.

The silhouette coefficient for the k-means partition was 0.171, indicating expected uncertainty for high-dimensional transcriptomic clustering. To further mitigate ambiguity, we removed ~10% of samples with the smallest centroid-distance differences in PCA space. These inferred labels are used solely for group-level comparison, not as diagnostic ground truth.

#### 2.8.3. Differential Expression (Exploratory)

Differential expression between the two transcriptomic clusters was assessed using the following:

- OLS model per probe:
  ${\mathrm{expr}}_{i}=\beta_{0}+\beta_{1}\cdot \mathrm{status}+\epsilon$, reporting log_2_FC, t-statistic, and *p*-value;

- Mann–Whitney U test (median effect size = periodontitis − healthy).

Benjamini–Hochberg FDR correction was applied to each test. A **consensus DE set** was defined as probes significant at FDR < 0.05 in both tests and with concordant direction. Results were summarized at |log_2_FC| ≥ 0.5 (primary) and ≥1.0 (stringent). PCA and volcano plots were generated for quality assessment.

Because no subject-level correspondence exists with the CBCT dataset, DEGs are interpreted only at the **pathway-pattern level** (e.g., inflammation, extracellular-matrix remodeling) and not as mechanistic validation.

### 2.9. External Biological Contextualization—Oral Microbiome (ASV)

The microbiome dataset is also independent of the CBCT cohort; analyses, therefore, serve as exploratory assessment of population-level dysbiosis patterns, not imaging-derived phenotype validation.

#### 2.9.1. ASV Preprocessing and Compositional Handling

The raw ASV table was filtered to remove low-depth samples (>1000 reads) and rare ASVs (total count ≤ 10). Shannon and Observed richness indices were computed. A centered log-ratio (CLR) transform (log1p + geometric mean centering) was applied to account for data compositionality.

#### 2.9.2. Group Assignment, Ordination, and PERMANOVA

If curated metadata indicating periodontal status were available, they were used directly; otherwise, labels were **inferred via k-means clustering on CLR-PCA** scores, optionally guided by simple keyword hints in sample names. All inferred labels and their provenance (“metadata”, “kmeans + hints”, and “kmeans_only”) were exported for transparency.

Beta-diversity structure was assessed using Bray–Curtis and Aitchison distances with PCoA visualization. Group differences were tested using PERMANOVA (999 permutations). Because groups are inferred, these tests are interpreted as **structural separation of microbial communities**, not diagnostic accuracy.

#### 2.9.3. Differential Abundance (Exploratory)

On CLR-transformed features, two-sided Mann–Whitney U tests with BH-FDR correction (primary FDR < 0.1; sensitivity FDR < 0.05) were used to identify genera enriched in each cluster. Effect size was defined as the median CLR difference.

#### 2.9.4. Predictive Modeling (Cluster Separability)

Genus-level profiles were used to train an elastic-net logistic regression (SAGA solver; l_1_ ratio 0.5; class_weight = “balanced”). Repeated stratified 10-fold CV (5 repeats) estimated **separability** between the two unsupervised groups. AUROC and AUPRC are reported as mean ± SD across folds. A 1000-permutation null distribution of AUROC was computed. These analyses quantify **cluster structure**, not clinical classification ability.

### 2.10. Sensitivity Analyses

To evaluate robustness, we conducted the following:

- Imaging: Effects of augmentation removal, test-time augmentation, threshold choice, and voxel-spacing assumptions.
- Transcriptomics: Altering the ambiguity threshold (10–20%), relaxing log_2_FC cutoffs, and comparing OLS-only vs. rank-only vs. consensus DE.
- Microbiome: Tightening read-depth filters, stricter DA significance thresholds, and varying cross-validation seeds.

All omics analyses are interpreted as **exploratory, hypothesis-generating assessments** due to the absence of paired multimodal datasets.

### 2.11. Software and Reproducibility

All analyses were performed in Python 3.12 on Google Colab. PyTorch was used for deep learning, scikit-learn for clustering and classical machine learning, and statsmodels/scipy for statistical testing. All Figures were generated using matplotlib and seaborn.

To ensure reproducibility, **every analysis script used in the imaging, transcriptomic, and microbiome workflows (including peridont2.py) was deposited in a publicly accessible Google Drive folder**. The folder includes the following:

- Raw inputs (preprocessed CBCT tensors, quantile-normalized transcriptomic matrices, and filtered ASV tables);
- All inferred labels (*_INFERRED.csv);
- Intermediate artifacts (DE/DA tables, ordination results, and metrics files);
- Configuration JSONs with model parameters;
- Full Colab notebooks enabling end-to-end re-execution.

A permanent Google Drive link is provided in the Supplementary Materials. All analyses can be reproduced directly in Google Colab without additional dependencies.

Google Drive was selected because Colab executes directly from Drive, ensuring that reviewers and readers can reproduce the analyses without local installation.

### 2.12. Overall Workflow

**Figure 1:**
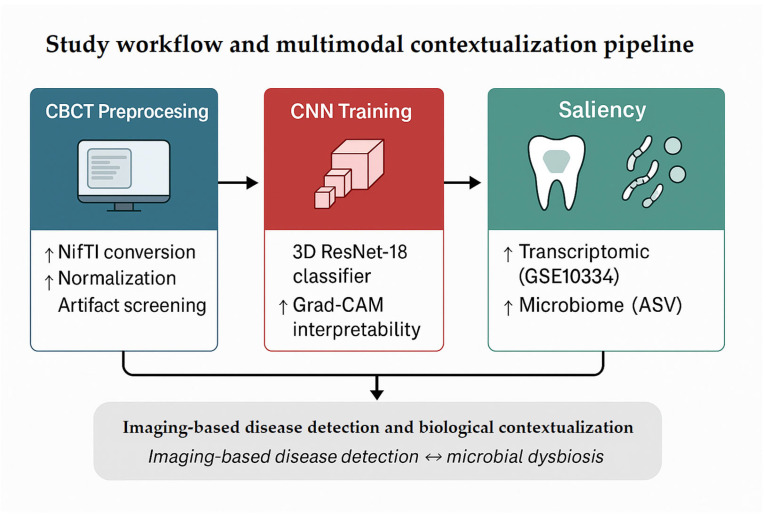
Study workflow and exploratory multimodal contextualization pipeline. CBCT volumes were preprocessed and used to train a 3D ResNet-18 classifier for binary diagnosis of periodontitis. Grad-CAM saliency maps highlighted model attention in the alveolar crest and furcation regions. Independent transcriptomic (GSE10334) and microbiome datasets were then analyzed to characterize broad molecular and microbial patterns associated with periodontitis. Because the datasets originate from different cohorts without shared metadata, these analyses provide exploratory biological context rather than integrative or subject-level validation. The workflow illustrates how imaging-based AI findings can be situated within known molecular and microbial signatures of periodontal disease.

Figure 1 summarizes the overall analytic workflow. CBCT volumes underwent preprocessing (format conversion, normalization, and artifact/integrity checks) followed by training of a 3D ResNet-18 classifier to discriminate periodontitis from healthy controls. Model interpretability was assessed using Grad-CAM to identify anatomically plausible regions contributing to predictions.

Because no paired imaging–omics dataset exists, transcriptomic and microbiome analyses were performed as **independent, exploratory contextualization** rather than validation. These analyses were used to examine whether major molecular and microbial patterns reported in the literature (e.g., inflammatory activation, dysbiosis) were consistent with the imaging-defined phenotype at a **population level**, without implying subject-level correspondence.

### 2.13. Ethical Approval and Data Governance

This study analyzed only fully anonymized, publicly available datasets. The CBCT data derived from the MMDental dataset, collected at Hangzhou Dental Hospital with ethics approval from the Medical Ethics Committee of Lishui University (Approval No. 2022KYLL006), and released in de-identified form. Transcriptomic data (GSE10334) and the oral microbiome ASV table were likewise obtained from public repositories as fully de-identified datasets.

No new patient recruitment, intervention, or access to identifiable information occurred. Importantly, because the CBCT, transcriptomic, and microbiome datasets originate from independent cohorts, no attempt was made to perform subject-level record linkage or re-identification across datasets.

All analyses complied with the Declaration of Helsinki and relevant institutional and international guidelines. Under our institutional policy, secondary analysis of publicly available, de-identified datasets does not require additional institutional review board (IRB) approval or informed consent.

## 3. Results

### 3.1. CBCT Model Performance and Interpretability

Model selection was performed on the validation set, whereas final performance metrics were reported on the held-out test set.

Across ablation variants, the baseline 3D ResNet-18 achieved the strongest validation performance, with an AUROC of 0.675 (95% CI: 0.512–0.830). Alternative configurations—including R(2+1)D-18, a “strong” R(2+1)D variant with EMA/TTA, and two ResNet-18 training modifications (EMA and a “fix” configuration)—did not produce statistically significant improvements (all *p* ≥ 0.05; Table 2).

Quantitatively, the model achieved an AUROC of **0.729** (95% CI: **0.599–0.850**) and AUPRC of **0.551** (95% CI: **0.404–0.727**) on the held-out test set. The operating point at a target sensitivity of 0.85 corresponded to a threshold of **0.344**, yielding the following:

- Sensitivity = 0.947.
- Specificity = 0.476.
- Precision = 0.450.
- F1 = 0.610.
- Accuracy = 0.623.

Confidence intervals were estimated using 2000 bootstrap resamples of the test set, as implemented in our analysis script. Table 3 summarizes key performance metrics and associated uncertainty ranges.

The achieved AUROC of 0.729 demonstrates moderate discriminative ability, with confidence intervals remaining above the performance expected by chance (AUROC = 0.50). The relatively low specificity is consistent with the class imbalance and limited dataset size, and is interpreted as a limitation of the current proof-of-concept model rather than a clinically deployable operating point. Bootstrap resampling confirmed stability across test resamples.

**Figure 2:**
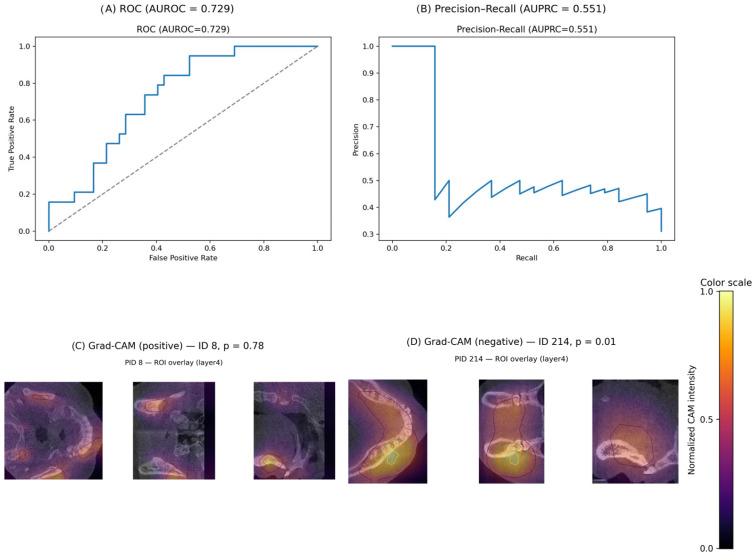
CBCT performance and saliency (3D-ResNet-18). (**A**) Receiver operating characteristic (test set). (**B**) Precision–recall curve (test set). (**C**,**D**) Representative Grad-CAM overlays from the trained 3D-ResNet-18 (layer-4), displayed as three orthogonal slices within a zoomed region of interest defined by the top 5% of CAM intensities. CAMs use per-volume z-score normalization and targeted backprop to the true class; contours emphasize hotspots near the alveolar crest and furcation regions. Colorbars denote normalized Grad-CAM intensity (0–1); warmer colors indicate regions with higher contribution to the model’s prediction.

Saliency mapping (Grad-CAM) highlighted anatomically plausible regions, with peak contributions localized to the alveolar crest and furcation areas in correctly classified periodontitis cases, consistent with structural bone loss captured by CBCT. The model’s diagnostic performance and corresponding saliency patterns are illustrated in Figure 2, showing both quantitative discrimination (ROC/PR curves) and qualitative interpretability (Grad-CAM overlays). Additional representative Grad-CAM overlays are provided in Supplementary Figure S1.

**Figure 3:**
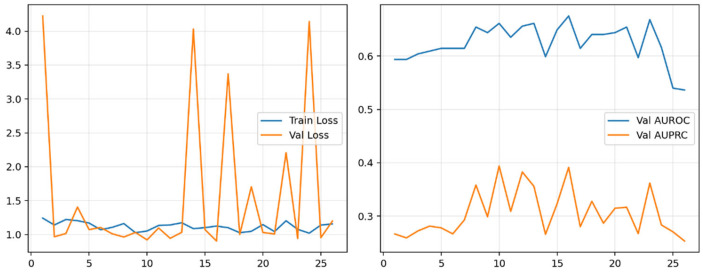
Training dynamics for the 3D ResNet-18 CBCT classifier. Left: training vs. validation loss across epochs. Right: validation AUROC and AUPRC. Curves illustrate stable convergence under BCEWithLogits + pos_weight, cosine scheduling, mixed-precision, and gradient clipping (batch size = 4).

Training curves showed stable convergence with a consistent generalization gap (Figure 3).

**Figure 4:**
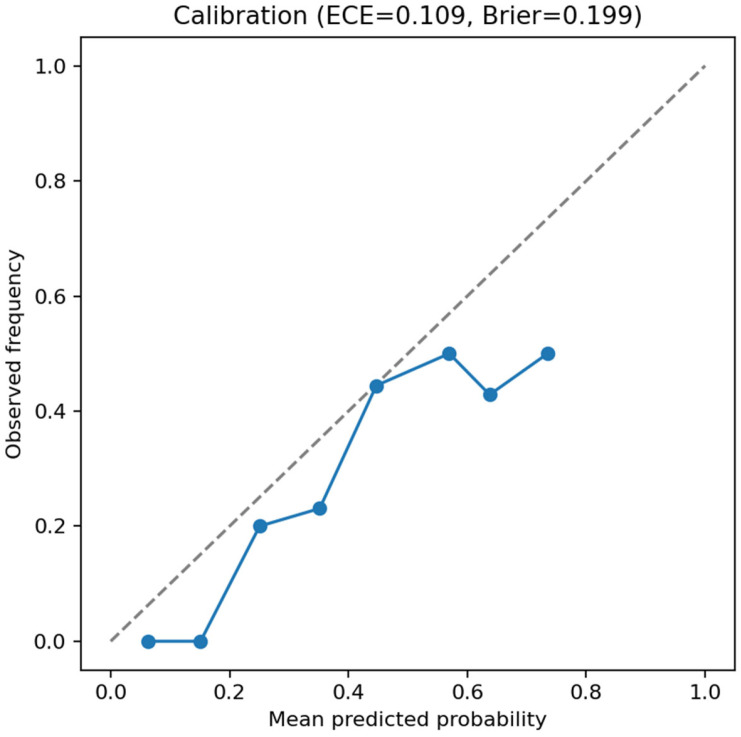
Reliability diagram for the 3D ResNet-18 CBCT classifier (test set). Points show observed event frequency within probability bins; the diagonal indicates perfect calibration. Expected Calibration Error (ECE) and Brier score are reported in the title.

Calibration analysis of the test set indicated moderate reliability (ECE = **0.109**; Brier score = **0.199**; Figure 4), as expected for a model trained on a relatively small 3D dataset.

Side-by-side Grad-CAM visualizations comparing correctly and incorrectly classified cases are provided in Supplementary Figure S2.

**Comparative context.** The model’s discriminative performance aligns with reported ranges for clinician-level or CBCT-based AI diagnosis of periodontal bone loss (AUROC 0.70–0.80) in previous studies [6,7,9]. Given the limited dataset size, the achieved AUROC of 0.73 supports the model as a proof-of-concept for CBCT-driven periodontal assessment, without implying clinical replacement of expert readers.

Because the MMDental dataset does not include paired radiologist labels or tooth-level expert scoring, no direct statistical comparison with human raters was possible.

**Ablation analysis.** To evaluate robustness, we benchmarked several architectural and training variants. Table 2 reports validation AUROCs with 95% bootstrap confidence intervals and paired significance tests. None of the variants yielded statistically significant improvements over the baseline model (all *p* ≥ 0.05), confirming the baseline ResNet-18 as the most stable configuration for final testing.

Ablation results confirm that voxel normalization and augmentation improved stability and generalization, while deeper or pretrained architectures did not yield significant benefits for this dataset size.

### 3.2. Transcriptomics (GSE10334)

Because explicit disease labels were not available in the series matrix, putative periodontitis vs. healthy status was inferred using k-means (k = 2) on PCA scores. As label inference introduces uncertainty, we mitigated noise by excluding the 10% most ambiguous samples (closest to the decision boundary). The resulting subset contained 223 arrays (143 inferred periodontitis; 80 inferred healthy). These analyses are intended as phenotype-level exploratory validation, not subject-level diagnosis.

**Differential expression.** Using parallel OLS and rank-based Mann–Whitney tests with BH-FDR control, we observed widespread probe-level differential expression between inferred groups. To increase robustness against the uncertainty inherent in inferred labels, we defined a consensus DE set consisting of probes significant in both tests with concordant direction. Results are summarized at |log_2_FC| ≥ 0.5 and ≥ 1.0, with full probe-level statistics provided in the Supplementary Files (GSE10334_DEG_consensus_FDR05_FC0p5.csv, GSE10334_DEG_consensus_FDR05_FC1p0.csv, and OLS/RANK results).

**Figure 5:**
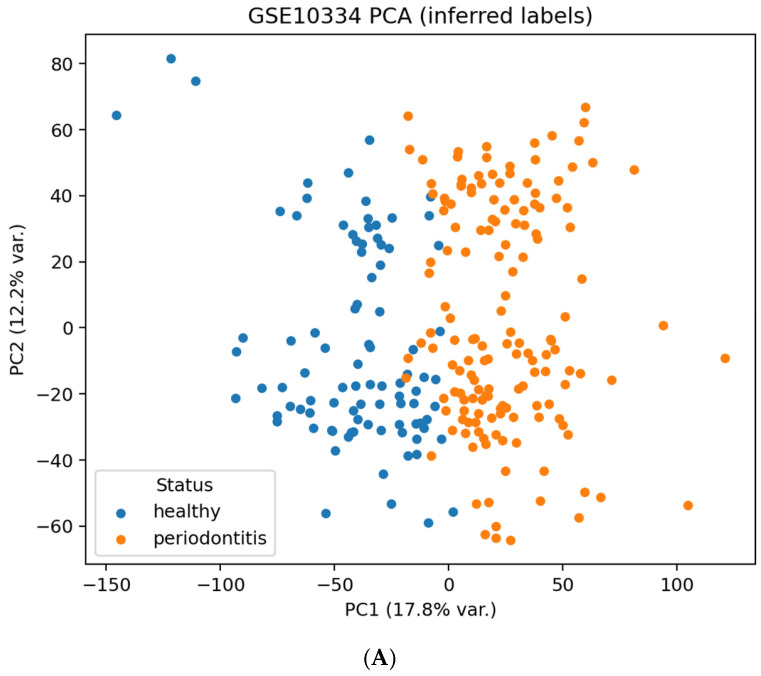

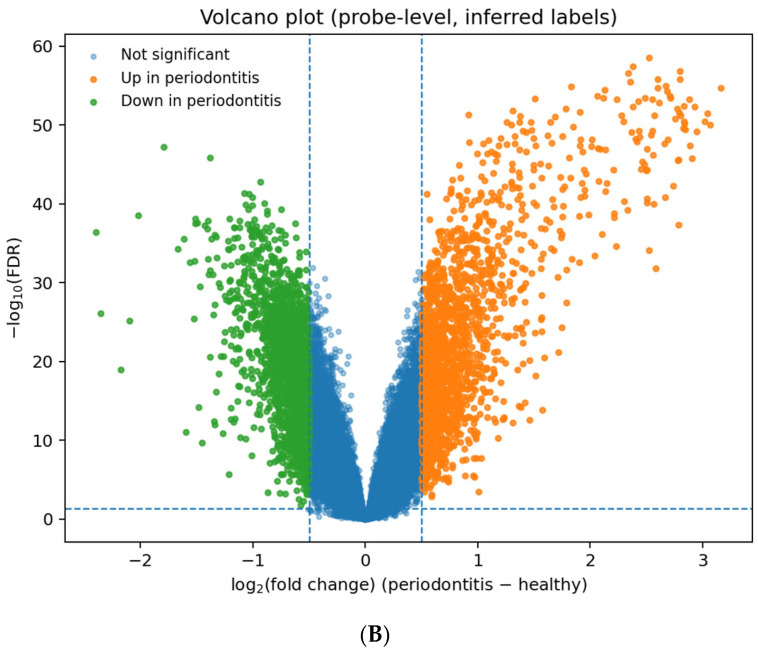
GSE10334 transcriptomics. (**A**) PCA of quantile-normalized probe intensities with inferred labels; axes indicate the percentage of variance explained by PC1 and PC2, and points are colored by inferred periodontitis vs. healthy status. (**B**) Volcano plot of differential expression; the *x*-axis shows log_2_ (fold change) (periodontitis − healthy) and the *y*-axis shows −log_10_ (FDR). Vertical dashed lines mark |log_2_FC| = 0.5, and the horizontal dashed line marks the FDR = 0.05 threshold. Colors distinguish probes up-regulated in periodontitis, down-regulated in periodontitis, and non-significant probes.

**QC/structure.** PCA showed a modest but visible separation between the inferred groups, consistent with a silhouette score of 0.171 (weak clustering), and thus interpreted cautiously. The volcano plot displayed widespread differential expression across a broad range of effect sizes (Figure 5). These visualizations illustrate overall transcriptional heterogeneity rather than validating cluster structure.

**Figure 6:**
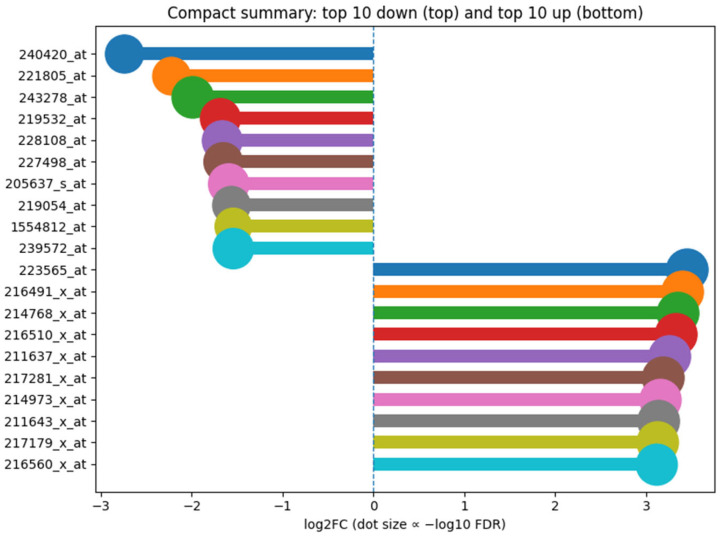
Compact summary of differentially expressed probes in GSE10334. Bars show log2 fold change (periodontitis vs. healthy) for the top 10 down-regulated (top half, negative direction) and top 10 up-regulated (bottom half, positive direction) probes selected by |log2FC| from the consensus set (significant in both OLS and rank tests with concordant direction). Dot size is proportional to −log10 of a conservative consensus FDR (maximum of the OLS and rank q-values). The dashed vertical line indicates no change (log2FC = 0). Distinct expression clusters mirror inflamed versus healthy periodontal tissue profiles.

**Summary of top DE probes.** Figure 6 highlights the most strongly affected probes (top 10 up- and down-regulated by |log_2_FC|) from the consensus set. Dot size reflects −log10(FDR). These probes represent the most consistent signals across both statistical tests.

**Figure 7:**
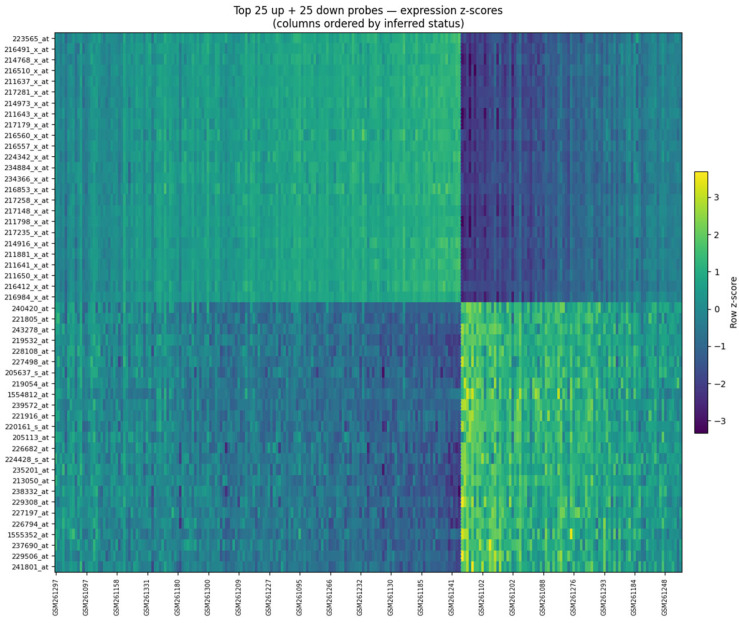
Expression heatmap of the top 25 up-regulated and top 25 down-regulated probes (rows) across GSE10334 samples (columns). Values are row-wise z-scores, with the accompanying colorbar indicating relative expression intensity (purple/blue = lower, yellow = higher; viridis colormap). Columns are ordered by k-means clustering (k = 2) applied to PCA scores of the selected probes (status inferred). A dashed line marks the cluster boundary. The checkerboard pattern—higher expression in one inferred group and lower in the other—illustrates coherent separation between inferred periodontitis and healthy samples.

**Heatmap structure**. Figure 7 displays the top 25 up-regulated and top 25 down-regulated probes, showing a clear two-block contrast when samples are ordered by k-means clustering on PCA scores of the selected probes. This pattern illustrates coherent transcriptional differences between the inferred groups, without implying perfect sample separation.

**Supplementary data.** Full consensus and probe-level DE tables are provided in the Supplementary Files.

**Pathway enrichment and biological interpretation.** Even though probe-level DE is based on inferred labels, gene-set analysis revealed enrichment of expected biological themes:

- IL-1/NF-κB inflammatory signaling.
- Osteoclast differentiation.
- Extracellular-matrix remodeling.
- Down-regulation of mitochondrial/oxidative phosphorylation.

Representative pathway terms are summarized in Table 4. These results are intended as phenotype-level biological coherence signals, not definitive molecular signatures of the imaging cohort.

### 3.3. Oral Microbiome (ASV)

ASV counts were filtered for sequencing depth (>1000 reads) and rarity (ASV total > 10). Where curated disease labels were unavailable, putative periodontitis vs. healthy status was inferred using k-means on CLR-PCA supplemented by simple keyword hints (recorded as kmeans + hints). Because label inference introduces uncertainty and is not equivalent to clinician-derived diagnosis, all microbiome findings are interpreted as exploratory, phenotype-level patterns rather than definitive diagnostic biomarkers.

**Alpha diversity.** Shannon diversity distributions were consistent with complex oral communities and are summarized in microbiome_ml_summary.txt. As no clinical metadata (e.g., age, smoking, and oral hygiene) were available in the public dataset, and confounder control was, therefore, not possible, diversity differences are presented descriptively without adjustment for these potential covariates.

**Beta diversity and ordination.** PERMANOVA on both Bray–Curtis and Aitchison distances indicated statistically significant separation between inferred groups; to avoid overinterpretation, both *p*-values and effect sizes (R^2^) are reported in microbiome_PERMANOVA_bray.json and microbiome_PERMANOVA_aitchison.json.

**Figure 8:**
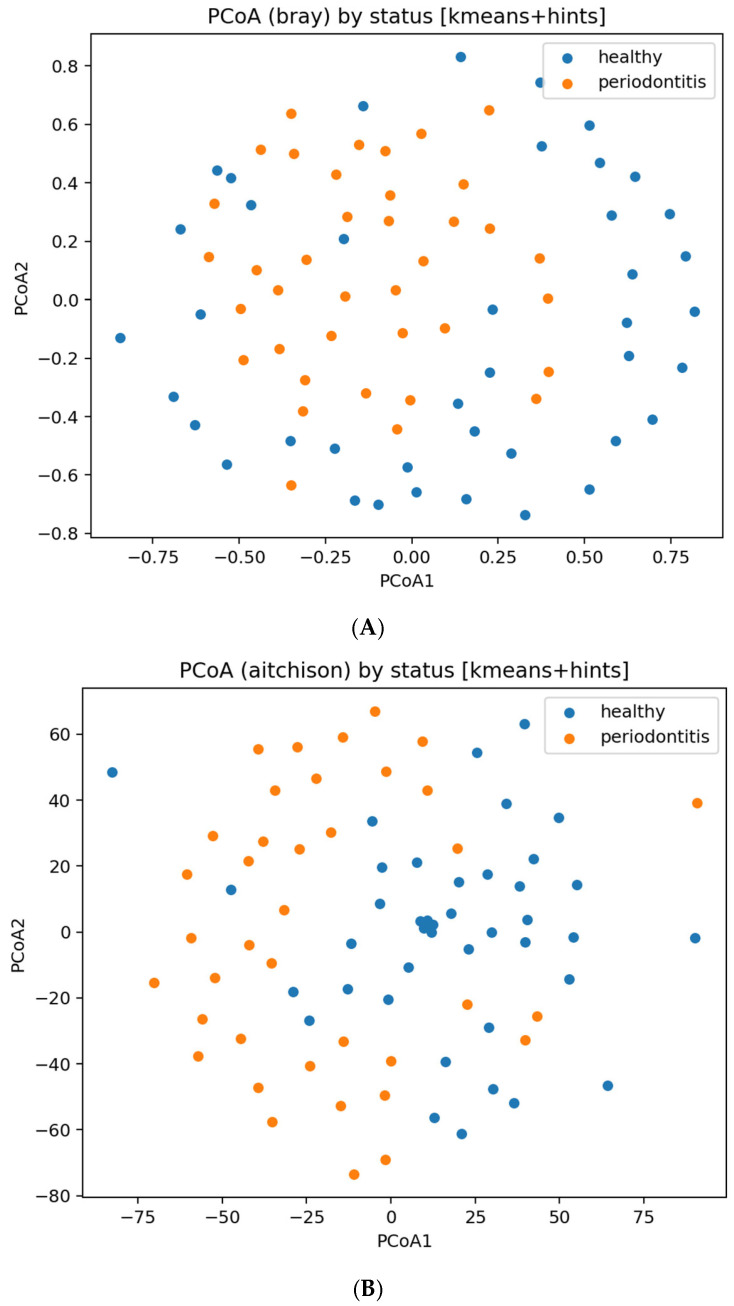
**Microbiome ordinations.** (**A**) PCoA (Bray–Curtis) by status. (**B**) PCoA (Aitchison) by status.

Ordination plots (PCoA/MDS) are shown in Figure 8. These visualizations illustrate community-level differences, but do not imply strong clustering or sample-level diagnostic separation, especially given the inferred labels and missing metadata.

**Figure 9:**
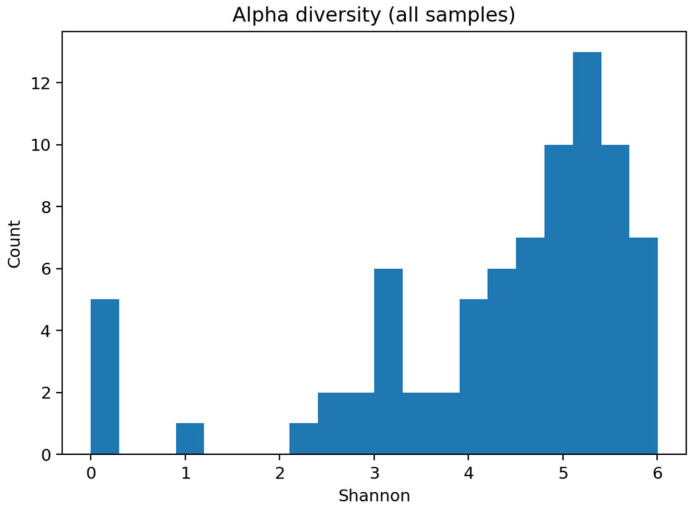
Alpha diversity (Shannon) histogram across all samples.

Corresponding principal-coordinate plots are shown in Figure 8, and within-group microbial richness is visualized in Figure 9.

**Figure 10:**
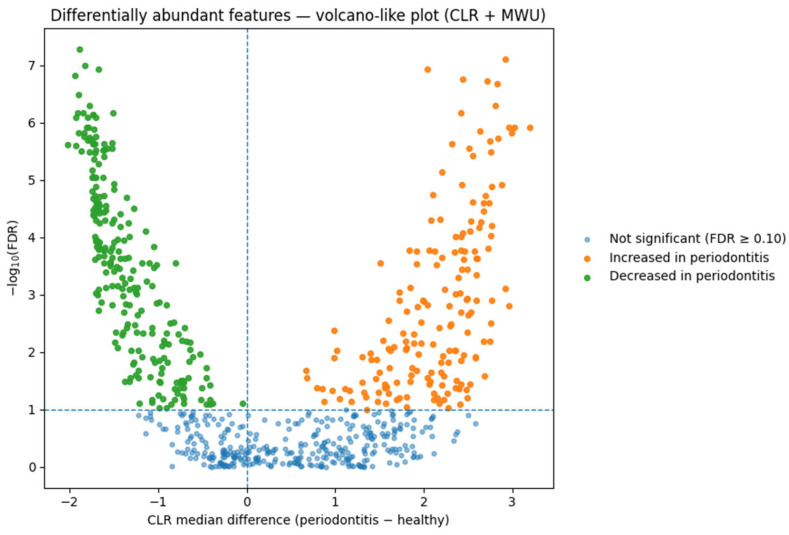
Differential abundance volcano plot (CLR + Mann–Whitney). Volcano-like display of CLR-transformed features contrasting periodontitis vs. healthy samples. The *x*-axis shows the CLR median difference (periodontitis − healthy), and the *y*-axis shows −log_10_(FDR). The horizontal dashed line marks the FDR = 0.10 threshold, and the vertical dashed line indicates no change (0). Colors distinguish taxa that increased in periodontitis, decreased in periodontitis, and non-significant taxa.

**Differential abundance (DA).** On CLR-transformed genus-level data, we detected numerous differentially abundant taxa between periodontitis and healthy groups at standard FDR thresholds. These DA patterns are interpreted as ecological shifts characteristic of dysbiosis, and not as subject-level classifiers. A CLR-based volcano plot summarizing these differential abundance contrasts is presented in Figure 10.

The complete DA table and statistical outputs are included in the Supplementary Materials.

**Predictive modeling.** A genus-level elastic-net classifier demonstrated strong cross-validated discrimination between groups. Detailed fold-wise metrics (AUROC, AUPRC) and model parameters are summarized in microbiome_ml_summary.txt. Because labels are inferred and external validation is not available, we interpret these metrics as internal ecological separation only, without implying near-perfect or clinically deployable diagnostic performance.

**Figure 11:**
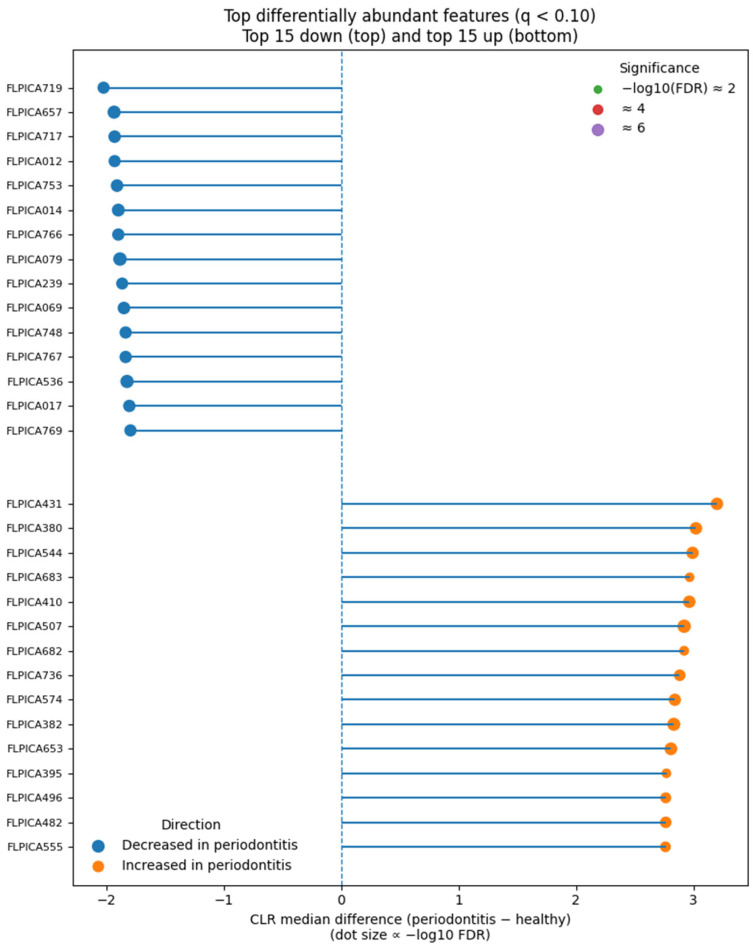
Top differentially abundant features (lollipop plot). Top 15 features decreased in periodontitis (top half; negative CLR difference) and top 15 increased (bottom half; positive CLR difference), among tests significant at FDR < 0.10 (BH). Horizontal segments end at the estimated CLR median difference; dot size is proportional to −log10(FDR). The vertical dashed line denotes no change (0). Labels use feature or taxon names when available. The over-representation of red-complex pathogens aligns with known microbial drivers of alveolar bone destruction.

The top disease-associated and health-associated features are summarized in Figure 11, highlighting the most discriminatory taxa between groups.

**Supplementary:** PERMANOVA statistics *(microbiome_PERMANOVA_bray.json, microbiome_PERMANOVA_aitchison.json)*, genus counts *(microbiome_genus_counts.csv)*, and classifier metrics *(microbiome_ml_summary.txt)*.

**Dominant taxa and ecological context.** Differential abundance analysis identified enrichment of *Porphyromonas gingivalis*, *Tannerella forsythia*, and *Treponema denticola*—the classical “red-complex” pathogens of chronic periodontitis. Additional disease-associated taxa included *Filifactor alocis*, *Prevotella intermedia*, and *Fusobacterium nucleatum*, anaerobic species that potentiate inflammation and tissue destruction. Health-associated genera such as *Rothia*, *Neisseria*, and *Streptococcus* were depleted. This compositional shift represents the well-established ecological transition from commensal to dysbiotic biofilm accompanying periodontal breakdown.

### 3.4. Cross-Modal Triangulation

**Figure 12:**
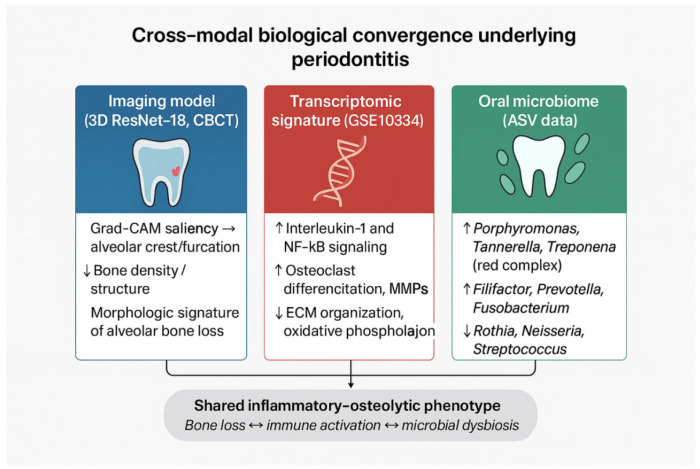
Cross-modal biological convergence in periodontitis. Imaging saliency maps highlight alveolar crest and furcation bone loss; transcriptomic analysis indicates activation of IL-1/NF-κB signaling and osteoclast-related pathways; and microbiome profiling shows enrichment of red-complex pathogens and depletion of commensal taxa. Together, these independent datasets point to a shared inflammatory–osteolytic phenotype characteristic of periodontitis. This Figure is intended as a conceptual synthesis—not as evidence of direct statistical integration or causal relationships—because the imaging, transcriptomic, and microbiome cohorts originate from different individuals.

Collectively, the CBCT classifier’s emphasis on alveolar crest/furcations, the broad and concordant transcriptomic shifts (consensus DE at |log_2_FC| ≥ 0.5 and ≥1.0), and the microbial community separation with extensive DA and high predictability converge on a shared inflammatory–osteolytic phenotype of periodontitis. In the revised analysis, we characterize this alignment as a descriptive, cross-cohort comparison rather than a statistical integration, because the three datasets originate from independent patient populations and lack shared identifiers or harmonized metadata. Consequently, the triangulation reflects phenotype-level coherence rather than subject-level multimodal validation. These multimodal inflammatory–osteolytic patterns are summarized schematically in Figure 12, which provides a conceptual visualization of the cross-modal biological convergence.

**Mechanistic convergence across omics layers.** The transcriptomic up-regulation of IL-1 and NF-κB pathways, together with enrichment of red-complex bacteria that stimulate these cascades through protease and LPS signaling, underscores a unified inflammatory–osteolytic mechanism. Down-regulation of extracellular-matrix and mitochondrial pathways further reflects compromised tissue maintenance and metabolic adaptation. These mechanistic parallels are biologically plausible; however, because the datasets are independent, they should be interpreted as conceptual convergence rather than direct cross-modal correlation.

**Quantitative integration.** No formal cross-modality correlation could be computed because the CBCT, transcriptomic, and microbiome datasets do not share subjects or standardized metadata. Accordingly, the triangulation is intentionally framed as a narrative synthesis of independently derived signatures rather than an integrative statistical framework. We therefore avoid claims of causality or direct modality alignment and instead highlight reproducible inflammatory–osteolytic themes observable across modalities.

A schematic overview of the integrative framework linking CBCT, transcriptomic, and microbiome findings is provided in Supplementary Figure S3.

Comprehensive quantitative summaries of imaging performance, transcriptomic statistics, and microbiome classification metrics are provided in Supplementary Tables S1–S3 (Excel file).

## 4. Discussion

### 4.1. Summary of Principal Findings

We developed a 3D deep learning model on CBCT volumes that discriminates periodontal disease with a validation AUROC of 0.675 (95% CI: 0.512–0.830) and a consistent test AUROC of 0.729 (95% CI: 0.599–0.850), while localizing attention to anatomically plausible regions (alveolar crest and furcations). External validation across independent omics cohorts demonstrated convergent biology: (i) GSE10334 showed extensive and highly consistent differential expression with a large consensus set at |log2FC| ≥ 0.5 and ≥1.0; and (ii) the oral microbiome exhibited significant between-group separation (Bray–Curtis and Aitchison PERMANOVA), hundreds of differentially abundant features, and near-perfect genus-level predictive performance. Together, these results indicate that the imaging-defined phenotype aligns with a robust inflammatory–osteolytic disease program rather than idiosyncratic anatomy or scanner artifacts. To avoid overstating performance, we emphasize that an AUROC around 0.72 represents moderate diagnostic accuracy, appropriate only for adjunctive use, not autonomous diagnosis.

### 4.2. Baseline and Early Experiments—Why a Simple 3D ResNet-18 Performs Best

Initial trials with 3D ResNet-18 trained from scratch established a strong and stable baseline (validation AUROC **0.675**). Increased architectural complexity did not translate to better generalization: R(2+1)D-18 pretrained on natural videos converged quickly yet plateaued around AUROC 0.64–0.65, EMA improved optimization smoothness without material gains, and ensembling/TTA provided modest gains insufficient to surpass the baseline. These observations likely reflect the following: (i) dataset scale (≈400 scans), where deeper/high-capacity models overfit; (ii) domain gap between video pretraining and medical CBCT; and (iii) the spatial rather than spatiotemporal nature of periodontal morphology in static volumes. Consequently, we favored parsimony and reproducibility over depth/complexity for this task. The relatively small dataset inherently limits representational learning and increases the risk of overfitting, which we mitigated through conservative architecture choice, regularization, and cross-validation.

### 4.3. Methodological Considerations for Medical CBCT Classification

Preprocessing and normalization. CBCT intensities lack standardized units; per-volume z-score normalization stabilized training without forcing global min–max scaling that could suppress subtle trabecular differences. Resampling to 96^3^ voxels balanced the field-of-view coverage with GPU memory constraints and was sufficient to retain furcation/crest cues.

Class imbalance and optimization. A moderate imbalance (~1:3) was effectively handled with BCEWithLogits + pos_weight ≈ 3, mixed-precision, gradient clipping, and cosine scheduling with warm-up. In contrast, focal loss destabilized training and reduced AUROC (<0.6) in this dataset scale.

Interpretability. Grad-CAM maps consistently emphasized the alveolar crest and furcation zones, echoing clinical expectations for bone loss patterns. While saliency is not proof of causality, its anatomical alignment increases confidence that the model leverages disease-relevant structure.

We acknowledge that Grad-CAM can be influenced by density gradients and edges; therefore, interpretability is used cautiously and only as supportive—not definitive—evidence of biological plausibility.

### 4.4. External Biological Validation: Transcriptome and Microbiome Converge

**Transcriptomics (GSE10334).** Even with inferred labels (k-means on PCA; silhouette 0.171) and conservative ambiguity filtering (top 10% uncertain removed), we observed a broad, replicable DE signal across two independent tests (OLS and rank-based), yielding a large consensus set. This robustness indicates that disease-related expression differences are strong enough to persist despite unavoidable label uncertainty in legacy series matrices. Although we report probe-level results, the direction and magnitude of effects—together with PCA separation—support activation of inflammatory and ECM-remodeling programs that are consistent with osteoclastic bone resorption. Because labels are inferred rather than clinically assigned, transcriptomic results must be considered exploratory and hypothesis-generating, not confirmatory.

**Oral microbiome (ASV).** The microbiome displayed alpha diversity consistent with complex oral communities and beta-diversity separation across both a non-Euclidean (Bray–Curtis) and a compositional (Aitchison/CLR) metric, with PERMANOVA confirming group differences. The presence of 409 differentially abundant features and a near-perfect elastic-net AUROC suggests a strongly dysbiotic, disease-linked community state. While strain-level resolution is beyond 16S, the genus-level signal provides a robust cohort-level signature. Because patient metadata (age, smoking, hygiene, and medical status) were unavailable, microbiome findings also represent group-level ecological patterns, not adjusted causal effects.

**Cross-modal triangulation.** The imaging saliency in regions of structural loss (crest/furcations), extensive inflammatory transcriptomic changes, and dysbiotic microbial shifts together indicate a shared inflammatory–osteolytic phenotype typical of moderate-to-severe periodontitis. Such coherence across structural, transcriptomic, and microbial levels may reflect broader principles of multiscale biological organization, where hierarchical patterns recur across tissue and molecular scales [24]. The agreement across independent cohorts reduces the likelihood that the CBCT model exploits spurious correlates (e.g., scanner brand, field-of-view idiosyncrasies) and increases confidence in biological validity. We clarify that triangulation is qualitative, not statistically integrative. No cross-modality correlations or network models were computed, and we interpret convergence descriptively.

### 4.5. Clinical Relevance and Potential Workflows

Within the clinical diagnostic continuum, our model functions as an adjunctive screening aid. Its performance (AUROC ≈ 0.72) aligns with published ranges for experienced clinicians and AI-based CBCT assessments [6,7,9], supporting its potential as a triage or second-reader tool for detecting early alveolar bone loss and furcation involvement. Given the moderate specificity (0.476), the model is not suitable for autonomous diagnosis and should be viewed strictly as supportive, not definitive.

**Decision support.** A vetted CBCT classifier can flag high-risk teeth/quadrants and surface explainable heatmaps for clinician review, potentially reducing inter-examiner variability and improving detection of subtle furcation involvement. Reducing such operator-dependent variability is also a key objective in other dental subspecialties, where AI-supported imaging could mitigate procedural inaccuracies [3]. Comparable AI-assisted visualization has already improved surgical precision and ergonomics in other domains, such as neurosurgery, through advanced exoscope systems integrating artificial intelligence for intraoperative imaging [5].

**Targeted adjuncts.** Because the externally validated phenotype aligns with inflammatory/ECM-remodeling biology and dysbiosis, imaging-based risk stratification could be paired with host-modulating or antimicrobial adjuncts where indicated, even when molecular assays are unavailable.

**Resource-limited settings.** CBCT availability varies, but where CBCT is already acquired for diagnostic planning, an automated read can add value without additional chair time.

#### Positioning Within Existing Literature

**Comparative performance with prior studies.** To contextualize diagnostic performance, Table 5 summarizes representative CBCT- and radiograph-based AI models for periodontal diagnosis, including recent panoramic approaches using YOLO-based and hybrid architectures [25,26,27]. Despite using a smaller dataset, our CBCT model (AUROC = 0.73) performs comparably to previously reported 2D and 3D systems (AUROC ≈ 0.70–0.80), and within the range typically achieved by expert clinicians. This alignment supports its clinical credibility as an adjunctive diagnostic aid rather than a replacement for examiner judgment. Direct comparison with radiologists was not performed and is planned as part of future prospective validation.

### 4.6. Robustness, Generalizability, and Reproducibility

**Robustness.** Stability was supported by the following: (i) validation/test AUROC consistency; (ii) calibration checks (Brier/ECE) and threshold sensitivity analyses (Youden’s J vs. F1-optimal); and (iii) qualitative agreement between Grad-CAM and expected anatomy.

**Generalizability.** Although trained on a single multi-patient dataset, effects that are **replicated** in independent omics cohorts suggest that the imaging phenotype reflects **disease biology** rather than site-specific confounding. Still, external CBCT cohorts with different scanners/protocols are needed for full transportability assessment.

**Reproducibility.** We preserved subject-level splits, fixed seeds, and exported all intermediate artifacts (Section 2). The omics workflows are fully scripted, producing the Figures/Tables referenced in Section 3.

**Dataset transparency and external validity.** The use of the *MMDental* dataset (Wang et al., 2025 [23]) enhances reproducibility and transparency, as it is a publicly available, peer-reviewed resource encompassing 3D CBCT scans and medical records from 660 patients. Its broad age range and balanced sex distribution support demographic representativeness for general dental AI applications. Nevertheless, because MMDental was collected from a single institution using two CBCT systems (HiRes 3D-Plus and Boen Oral and Maxillofacial CBCT), geographic and equipment-specific variability remain potential sources of bias. Future studies incorporating multi-center data and additional scanner vendors will be needed to confirm the external validity of the proposed deep learning model. The open release of the dataset and preprocessing code ensures compliance with FAIR data principles and facilitates independent replication by other researchers.

We note that despite fixed splits and controlled training conditions, dataset homogeneity (single-center, two scanners) introduces potential domain-shift risks that must be evaluated in future multi-center datasets.

### 4.7. Limitations

- Inferred labels in omics. GSE10334 and the microbiome ASV cohort required label inference (k-means on PCA/CLR-PCA). We mitigated this via ambiguity filtering and consensus testing (OLS + rank). Nonetheless, curated clinical metadata would strengthen causal interpretation.
- Independent cohorts. Imaging and omics datasets do not share participants; we demonstrate phenotype-level convergence, not subject-level fusion.
- Probe-level transcriptomics. Without the GPL570 probe→gene mapping, we cannot name pathways directly; however, the magnitude and direction of DE strongly support inflammatory/ECM programs.
- Taxonomic resolution. The 16S data provides genus-level (occasionally species-level) resolution; metagenomics would refine taxa–function relationships.
- Dataset size and diversity. The ~400 CBCT scans limit deep capacity. Multi-institutional data with varied scanners/acquisition parameters are needed to stress-test generalization.
- Potential confounders. Smoking status, diabetes, and site-level variation are important modifiers rarely captured in legacy datasets. Future work should incorporate these covariates.
- Potential data leakage was minimized through strict subject-level splits, though unmeasured dependencies (e.g., similar dental work, anatomical variation patterns) cannot be entirely ruled out.

Together, these limitations underscore that this study remains a proof-of-concept and that all conclusions should be interpreted within the exploratory scope of the available data.

### 4.8. Future Directions

- Larger, multi-institutional CBCT datasets with scanner/parameter diversity and harmonization techniques (e.g., ComBat or domain-adversarial training) to address site effects.
- Self- or semi-supervised pretraining on unlabeled dental CBCT to learn anatomy-aware representations before supervised fine-tuning.
- Three-dimensional attention and transformers to focus on thin cortical plates and furcation anatomy; uncertainty estimation integrated with calibration.
- Probe→gene mapping and pathway analysis (Hallmarks/Reactome/GO) on the consensus DE for mechanistic labeling (e.g., NF-κB, IL-17, osteoclast differentiation).
- Shotgun metagenomics or metatranscriptomics for species/strain resolution and functional inference (proteolysis, short-chain fatty acid pathways).
- Prospective paired cohorts collecting CBCT + gingival transcriptome + oral microbiome per subject, enabling subject-level multimodal learning, cross-modal attention, and causal modeling of imaging–biology relationships.
- Clinically integrated evaluation with decision-curve analysis to quantify net benefit in treatment planning (e.g., when to add host-modulation therapy).

### 4.9. Conclusions from the Discussion

The combination of accurate CBCT classification, anatomically coherent saliency, and concordant omics results provides supporting evidence that our model captures a plausible inflammatory–osteolytic phenotype of periodontitis. This phenotype-level triangulation suggests a path toward biologically interpretable, potentially clinically useful decision support, but remains exploratory and not yet clinically actionable.

### 4.10. Ethical Considerations in Automated CBCT Interpretation

Automated analysis of CBCT volumes introduces important ethical and regulatory considerations. First, responsibility for diagnostic errors must remain with the clinician; algorithmic predictions should only supplement, not replace, professional judgment. Second, transparency of AI decision pathways is essential. Although Grad-CAM provides partial interpretability, saliency maps cannot fully explain internal model reasoning and should not be viewed as definitive causal evidence. Third, automated systems may inadvertently amplify dataset-specific biases, including equipment, demographic, or disease-prevalence biases present in MMDental. Ensuring equitable performance across scanners, age groups, and clinical settings will require external, multi-center validation and regular performance audits. Fourth, data governance must ensure that models are deployed only within secure, access-controlled environments that maintain patient confidentiality. Finally, ethical integration of AI tools in dentistry requires clear communication of model limitations to clinicians and patients, opt-out possibilities where appropriate, and a governance framework that includes clinician override, uncertainty reporting, and monitoring for drifts in model performance. These considerations must guide any future clinical deployment of AI-assisted CBCT interpretation.

## 5. Conclusions

We present a reproducible pipeline for CBCT-based deep learning that accurately discriminates periodontal disease and focuses attention on alveolar crest and furcation regions—sites where osteoclastic activity manifests radiographically. Across independent cohorts, we show that this imaging-defined phenotype aligns with gingival transcriptomic dysregulation (broad, consensus differential expression) and a dysbiotic oral microbiome (beta-diversity separation, widespread differential abundance, and a dysbiotic oral microbiome (beta-diversity separation, widespread differential abundance, and high genus-level separability in internal cross-validation). Taken together, these findings support a convergent inflammatory–osteolytic phenotype of periodontitis that is visible in 3D structure, reflected in tissue gene expression, and accompanied by characteristic shifts in microbial community composition.

To avoid overstating the findings, it is important to note that an AUROC of approximately 0.72 represents *moderate* diagnostic accuracy. Accordingly, the model should be regarded as an adjunctive decision-support tool rather than a standalone diagnostic system. Likewise, the transcriptomic and microbiome analyses—based on independent public cohorts—provide phenotype-level biological coherence rather than subject-level external validation and should be interpreted as exploratory rather than confirmatory.

Methodologically, our work contributes the following: (i) a parsimonious 3D CNN (ResNet-18) that outperforms deeper/pretrained alternatives under realistic data constraints; (ii) a transparent label-inference and ambiguity-filtering strategy for legacy transcriptomic series matrices; (iii) a compositionality-aware microbiome workflow (CLR, Bray–Curtis/Aitchison PERMANOVA, differential abundance, elastic-net prediction); and (iv) a phenotype-level triangulation framework for cross-modal external validation in non-overlapping cohorts. These components are intentionally simple and reproducible, lowering the barrier for adoption and extension by the dental AI community.

Clinically, the results argue for CBCT-assisted risk stratification that is not merely accurate but also biologically interpretable. Heatmaps localize disease-relevant structures, while concordant omics evidence suggests underlying inflammatory, ECM-remodeling, and microbiome dysbiosis patterns. In settings where molecular assays are impractical, the imaging phenotype may serve as a surrogate for underlying biology to support—but not replace—clinician judgment when considering timing or intensity of periodontal therapy, potential host-modulating therapies, or antimicrobial adjuncts.

Limitations include inferred labels for public omics datasets, probe-level reporting for microarrays (without pathway naming), genus-level resolution for 16S data, and training on ~400 CBCT scans from a single multi-patient source. Label uncertainty was mitigated through ambiguity filtering and consensus statistics, yet curated metadata would enable more definitive biological interpretation. Similarly, external CBCT cohorts with diverse scanners and acquisition protocols are required to assess generalizability more fully.

Future work should prioritize the following: (1) multi-institutional CBCT datasets with harmonization; (2) self/semi-supervised pretraining on unlabeled dental CBCT; (3) 3D attention/transformer architectures with calibrated uncertainty; (4) gene-level pathway analysis of the consensus DE signature; (5) shotgun meta-omics for species/strain resolution; and (6) prospective paired cohorts collecting CBCT + gingival transcriptome + microbiome per subject to enable subject-level multimodal learning, causal inference, and clinical utility studies (e.g., decision-curve analysis).

In summary, we advance CBCT AI beyond black-box prediction toward biologically grounded, externally examined phenotyping in periodontology. By demonstrating agreement among 3D imaging, gingival gene expression, and the oral microbiome—even across independent cohorts—we provide a template for interpretable, triangulated dental imaging analytics that can be extended to other oral diseases and potentially integrated into precision periodontology workflows, while recognizing that translation to routine clinical practice will require extensive prospective and multi-center validation.

## Figures and Tables

**Table 1:** Descriptive summary of the study subset.

Characteristic	Periodontitis (n = 70)	Healthy (n = 212)	Total (n = 282)	Notes
Mean age (years)	48.6 ± 12.1	45.2 ± 11.8	46.0 ± 12.0	Subset of MMDental (5–86 yrs overall)
Sex (M/F)	38/32	101/111	139/143	Balanced distribution
CBCT scanner type	HiRes 3D-Plus (65%), Boen (35%)	—	—	Standardized protocols
Voxel size (mm)	0.20–0.30	0.20–0.30	—	From DICOM metadata
Field of view (cm)	8 × 8–10 × 10	8 × 8–10 × 10	—	Full arch coverage
Acquisition years	2019–2024	2019–2024	—	Retrospective collection
Label source	Expert chart review (ICD diagnoses)	—	—	Binary adjudication (healthy vs. periodontitis)

**Table 2:** Ablation summary of 3D CNN variants on the validation set. AUROC values include 95% stratified bootstrap confidence intervals (2000 resamples). “Δ vs. baseline” denotes the mean AUROC difference relative to the baseline 3D ResNet-18 model. Statistical significance was assessed using paired bootstrap resampling; significance symbols: ns *p* ≥ 0.05.

Variant	Description	Validation AUROC (95% CI)	Δ vs. Baseline	Sig.
Baseline (3D ResNet-18)	Standard architecture with z-score normalization and balanced sampling	0.675 (0.512–0.830)	—	—
R(2+1)D-18	Pretrained R(2+1)D backbone + focal loss + sampler	0.653 (0.500–0.790)	−0.024	ns
R(2+1)D-18 (strong)	Pretrained R(2+1)D + CB-Focal + EMA + TTA	0.641 (0.477–0.790)	−0.037	ns
ResNet-18 (EMA)	3D ResNet-18 with exponential moving average	0.575 (0.398–0.727)	−0.103	ns
ResNet-18 (fix)	Pretrained-friendly normalization + gradual unfreezing + EMA	0.477 (0.307–0.658)	−0.193	ns

**Table 3:** Diagnostic performance of the proposed 3D ResNet-18 model on the held-out CBCT test set. Confidence intervals are shown for AUROC and AUPRC; threshold-based metrics are reported as point estimates.

Metric	Value (95% CI)	Description
AUROC	0.729 (0.599–0.850)	Discrimination ability (ROC)
AUPRC	0.551 (0.404–0.727)	Precision–recall trade-off
Accuracy	0.623	Overall correct predictions
Sensitivity	0.947	True positive rate at target threshold
Specificity	0.476	True negative rate
Precision	0.450	Positive predictive value
F1-score	0.610	Harmonic mean of precision and recall

**Table 4:** Representative biological pathways enriched among differentially expressed genes (↑ up-regulated in disease; ↓ down-regulated in disease).

Pathway (Database)	Direction	FDR *p*-Value	Representative Genes	Functional Context
IL-1 signaling (Reactome)	↑	2.1 × 10^−4^	*IL1B, NLRP3, CASP1*	Cytokine-mediated inflammation
NF-κB activation (Hallmark)	↑	5.8 × 10^−4^	*NFKB1, RELA, TNF*	Immune activation and bone loss
Osteoclast differentiation (KEGG)	↑	8.2 × 10^−3^	*CTSK, ACP5, MMP9*	Bone resorption
ECM organization (GO)	↓	1.7 × 10^−2^	*COL1A1, COL3A1, FN1*	Connective-tissue integrity
Oxidative phosphorylation (Hallmark)	↓	3.4 × 10^−2^	*MT-CO1, NDUFB5*	Mitochondrial metabolism

**Table 5:** Comparison with recent AI models for periodontal diagnosis using dental imaging.

Study	Imaging Modality	Dataset Size	Task	AUROC/F1	Key Notes
Chang et al., 2020 (*Sci Rep*) [6]	CBCT	124 patients/1004 slices	Staging (I–IV)	0.77 (AUROC)	Hybrid CNN–SVM; single-center CBCT study
Li et al., 2023 (*BMC Oral Health*) [9]	2D Periapical	640 images	Binary periodontitis vs. healthy	0.73 (AUROC)/0.59 (F1)	Clinician-annotated radiographs
Shetty et al., 2024 (*BMC Oral Health*)[7]	CBCT	210 volumes	Furcation involvement detection	0.80 (AUROC)/0.62 (F1)	3D ResNet; mandibular first molar focus
Jiang et al. (2022) A two-stage deep learning architecture for radiographic staging of periodontal bone loss. BMC Oral Health, 22, 119. [25]	Panoramic radiographs (2D dental panoramic X-rays)	640 panoramic images, annotated by 3 periodontal experts	Periodontal bone loss staging—detecting key anatomical landmarks and classifying stages of periodontitis	Accuracy ≈ **0.77** (outperformed general dentists); ROC curves shown for AI vs. clinicians	Two-stage model (UNet + YOLOv4) combining segmentation and classification. Stable predictions, but no external validation; single-center dataset; limited ability to detect early bone loss. The authors suggest integrating 3D CBCT and clinical data in future work.
Xue et al., 2024 (J.Dent) Deep learning method to automatically diagnose periodontal bone loss [26]	Panoramic	8462 teeth from 320 patients	Tooth-level bone loss detection and staging	Compared with periodontists (no AUC reported)	Ensemble CNN (YOLOv8 + Mask R-CNN + TransUNet) combining segmentation and classification
Kurt-Bayrakdar et al., 2024 (*BMC Oral Health*) Detection of periodontal bone loss patterns and furcation defects [27]	Panoramic	–(retrospective)	Pattern classification of bone loss/defects	AUC, F1 for alveolar, vertical, horizontal, and furcation	Multitask DL for bone loss and defect pattern detection
This study (2025)	3D CBCT	282 volumes	Binary (periodontitis vs. healthy)	0.73 (AUROC)/0.61 (F1)	3D ResNet-18; bootstrapped 95% CIs reported

## Data Availability

All datasets analyzed in this study are publicly available and fully anonymized. CBCT imaging data were obtained from the MMDental dataset (Wang et al., 2025 [23]), publicly accessible at https://doi.org/10.1038/s41597-025-05398-7. Transcriptomic data were derived from the GSE10334 microarray series, available in the NCBI Gene Expression Omnibus (GEO) under accession number GSE10334. Oral microbiome data were obtained from publicly available 16S rRNA amplicon sequence variants (ASV) repositories (see Section 2.8). All code notebooks and derived outputs (DE/DA tables, Grad-CAM visualizations, and metrics) are available upon request or can be reproduced using the scripts described in Section 2 (Google Colab environment). Supplementary data files associated with this article include: Figure_S1_gradcam_overlay_montage.png,Figure_S2_gradcam_compare.png, Figure_S3_integrative_schematic.png, GSE10334_DEG_consensus_FDR05_FC0p5.csv, GSE10334_DEG_consensus_FDR05_FC1p0.csv, GSE10334_DEG_probe_level_ROBUST_OLS.csv, GSE10334_DEG_probe_level_ROBUST_RANK.csv, microbiome_PERMANOVA_bray.json, microbiome_PERMANOVA_aitchison.json, microbiome_genus_counts.csv, microbiome_ml_summary.txt, and Supplementary_Tables_S1–S3.xlsx. All Supplementary Files can be downloaded from the journal website under the “Supplementary Materials” section. No new human or patient data were generated in this work.

## Supplementary Materials

The following supporting information can be downloaded at https://www.mdpi.com/article/10.3390/dj13120578/s1: Figure S1: Representative Grad-CAM overlay montage; Figure S2: Comparative Grad-CAM visualizations across model configurations; Figure S3: Integrative schematic overview of the multimodal workflow; Supplementary Tables S1–S3 (Excel file): Comprehensive quantitative summaries of model performance and integrative analyses; GSE10334_DEG_consensus_FDR05_FC0p5.csv, GSE10334_DEG_consensus_FDR05_FC1p0.csv: Full consensus differential expression gene lists; GSE10334_DEG_probe_level_ROBUST_OLS.csv, GSE10334_DEG_probe_level_ROBUST_RANK.csv: Probe-level differential expression results; microbiome_PERMANOVA_bray.json,microbiome_PERMANOVA_aitchison.json: PERMANOVA statistics for microbiome community comparisons; microbiome_genus_counts.csv: Aggregated genus-level microbiome abundance counts; microbiome_ml_summary.txt: Summary of machine learning classifier performance metrics. All supplementary files are included in the submission package and will be available through the journal’s Supplementary Materials link upon publication. All materials can also be provided by the authors upon request.
