# Supplementary figures and images for: Deep Learning Analysis of CBCT Images for Periodontal Disease: Phenotype-Level Concordance with Independent Transcriptomic and Microbiome Datasets
Source: Dent J (Basel). 2025 Dec 3;13(12):578. doi: 10.3390/dj13120578 (PMC12731456; doi:10.3390/dj13120578)

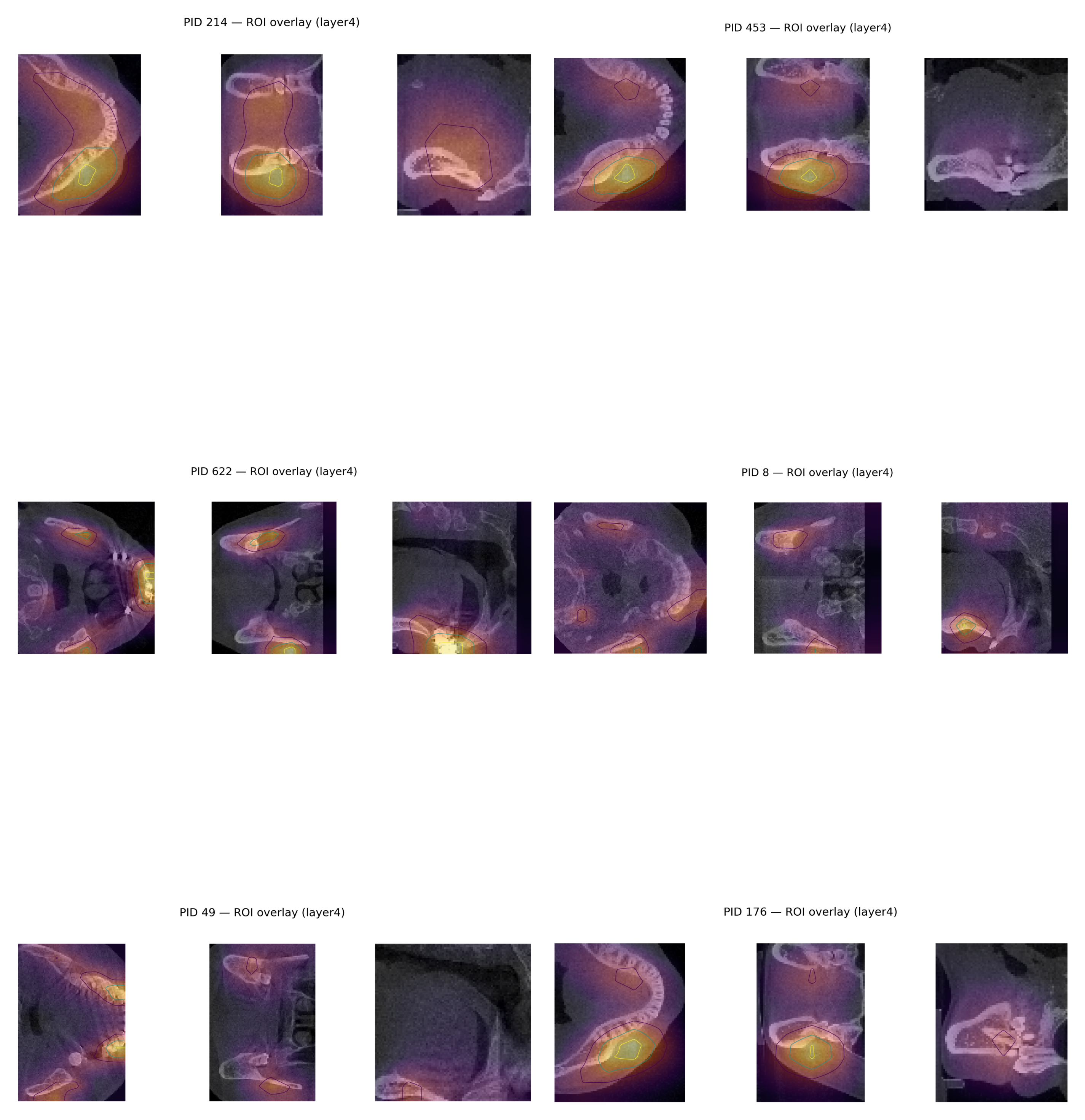

Supplement: Supplementary file 1 [file dentistry-13-00578-s001.zip › Figure_S1_gradcam_overlay.png]

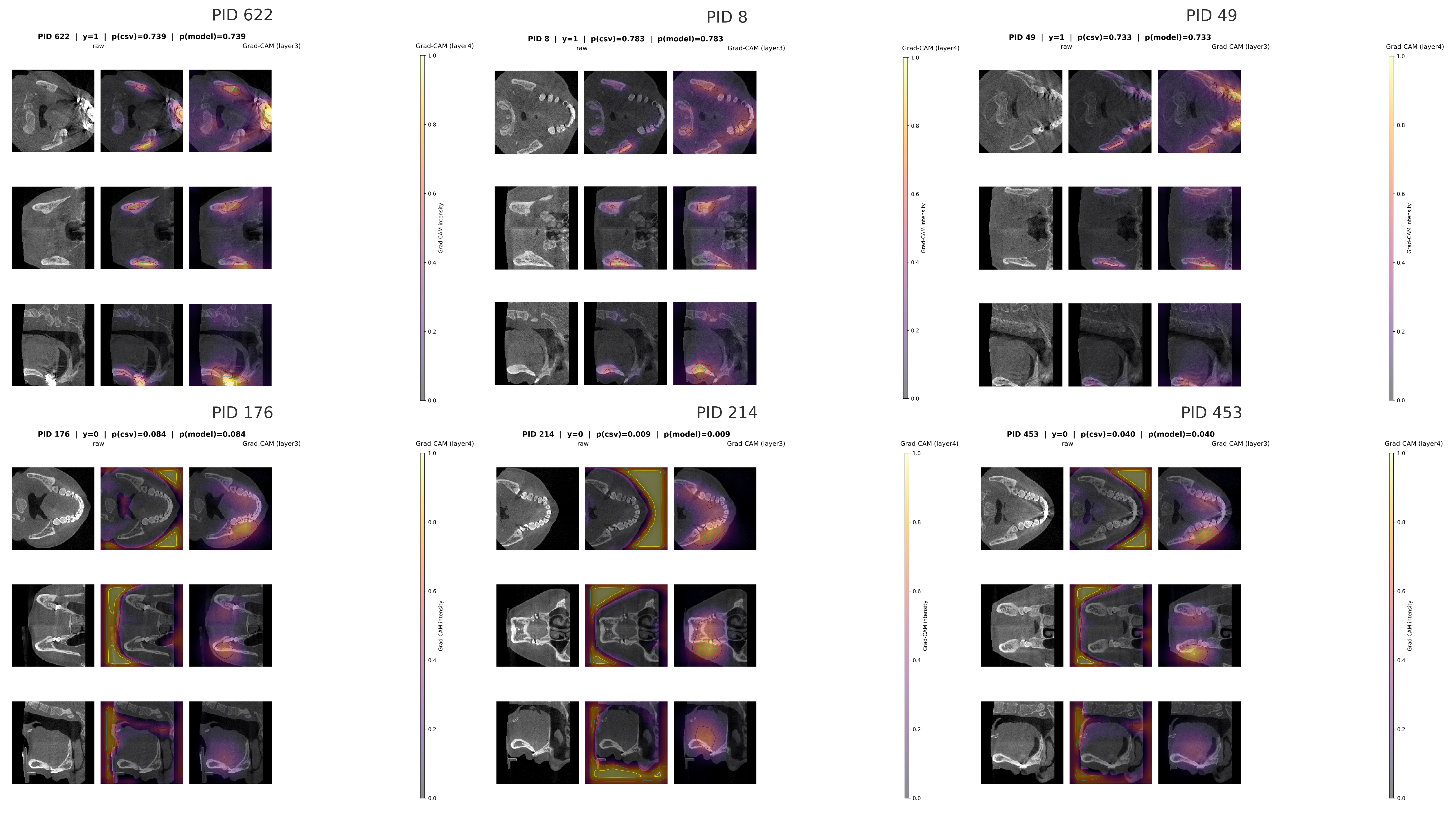

Supplement: Supplementary file 1 [file dentistry-13-00578-s001.zip › Figure_S2_gradcam_compare.png]

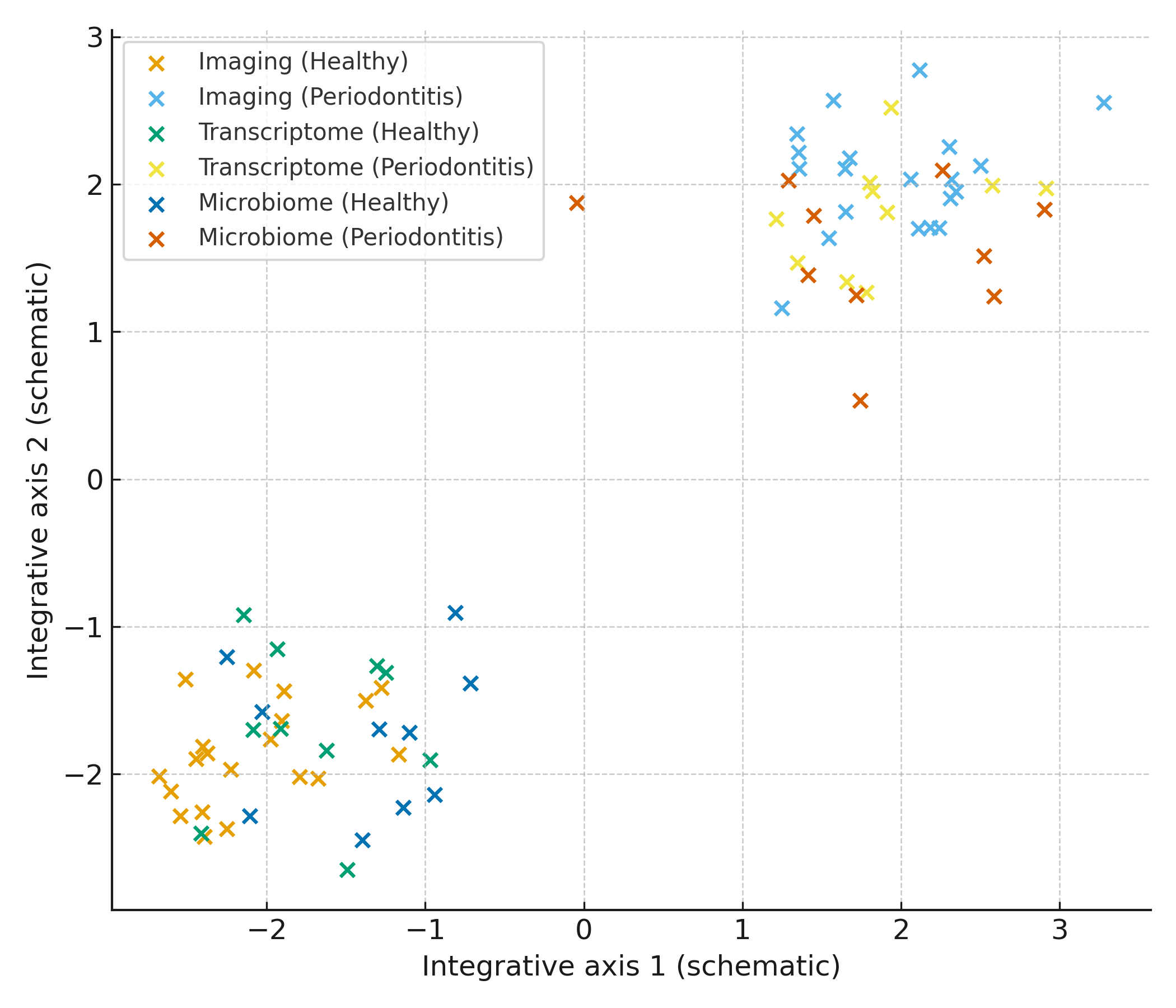

Supplement: Supplementary file 1 [file dentistry-13-00578-s001.zip › Figure_S3_integrative_schematic_no_title.png]
